# NLRP3 Inflammasome: A Potential Alternative Therapy Target for Atherosclerosis

**DOI:** 10.1155/2020/1561342

**Published:** 2020-03-31

**Authors:** Yang Liu, Chao Li, Honglin Yin, Xinrong Zhang, Yunlun Li

**Affiliations:** ^1^Experimental Center, Shandong University of Traditional Chinese Medicine, 250355 Ji-nan, China; ^2^Affiliated Hospital of Shandong University of Traditional Chinese Medicine, 250000 Ji-nan, China

## Abstract

Atherosclerosis (AS) is a complex and chronic inflammatory disease that occurs in multiple systems of the human body. It is an important pathological basis for a variety of diseases and a serious threat to human health. So far, many theories have been formed to explain the pathogenesis of atherosclerosis, among which “inflammation theory” has gradually become a research focus. This theory presents that inflammatory response runs through the whole progress of AS, inflammatory cells play as the main executors of AS, and inflammatory mediators are the key molecules of AS. In the inflammatory process of atherosclerosis, the role of NLRP3 in the atherosclerosis has gradually got the attention of researchers. NLRP3 is a kind of signal-transductional pattern recognition receptors (PRRs). After recognizing and binding to the damage factors, NLRP3 inflammasome will be assembled to activate IL-1*β* and caspase-1 pathways, resulting in promoting the inflammation process of AS, reducing the stability of the plaques, and finally increasing the incidence of adverse cardiovascular events. Taken above, the article will review the potential benefits of drugs targeting the NLRP3 inflammasome in the therapy of AS.

## 1. Introduction

Atherosclerosis (AS) is a chronic disease caused by many factors, which often causes some important adverse cardiovascular and cerebrovascular events, including coronary artery, carotid artery, cerebral artery-related diseases, and peripheral artery diseases. The prevalence of atherosclerosis is increasing year by year all over the world, which continues to threaten human health and make our society carry great burden [1].

The injury of the arterial intima and formation of lipid stripe are considered as the initial manifestation of atherosclerosis. Excessive low-density lipoprotein (LDL) accumulates and deposits in vascular subcutaneous tissue, which activates the immune stress of arterial endothelial cells and causes a series of inflammatory reactions [2]. LDLs are modified to oxidized LDLs (ox-LDLs), which stimulate endothelial cells to generate a large number of chemokines and recruit T cells and monocytes [3, 4]. Monocytes begin to bind with E-selectin and P-selectin at the activated endothelium and migrate to the intima. Vascular endothelial cells (VECs) secrete a variety of cytokines and chemokines to promote the migration of monocytes. The vascular endothelial monocytes are transformed into macrophages. Macrophages engulf lipids and form foam cells, which in turn promotes the occurrence and development of AS. With the development of atherosclerosis, lipid stripes gradually mature into fibrous plaques covered with the fibrous cap. During the formation of fibrous cap, due to the common influence of a variety of cytokines and chemokines, vascular smooth muscle cells (VSMCs) change from static and contractile state to active and synthetic state, and then move to the artery intima [5].

Inflammatory factors run through the whole process of atherosclerosis. In 1999, Ross proposed the hypothesis of inflammatory response to atherosclerosis [6]. Since then, more and more research has proved that inflammatory factors and inflammasomes play a critical role in the inflammatory response of atherosclerosis. Among them, the NLRP3 inflammasome which is a representative is paid much attention. NLRP3 inflammasome is related to many diseases involved in multiple systems, such as chronic obstructive pulmonary disease [7, 8], asthma [7], gout [9, 10], Crohn disease [11], heart failure [12], and myocardial infarction [13, 14]. Recently, the results of the CANTOS test [15] showed that the anti-inflammatory treatment aiming at the interleukin-1*β* (IL-1*β*) pathway significantly reduced the recurrence rate of cardiovascular events, which had nothing to do with lipid level. It directly confirms the theory of inflammation in atherosclerosis and provides a theoretical basis for the clinical anti-inflammatory treatment of atherosclerosis [15]. This review focuses on the role and regulatory mechanism of the NLRP3 inflammasome in atherosclerosis, and the NLRP3 inflammasome could be considered as a potential therapeutic target for AS.

## 2. NLRP3 Inflammasome

### 2.1. Structure of NLRP3

The NOD-like receptors (NLRs), as signal-transductional PRRs, are distributed in the cytoplasm and are important receptors in the signaling pathway against intracellular pathogens and injury factors. So far, 23 NLRs have been found in humans and 34 in mice.

NLR consists of three domains, with leucine-rich repeats (LRR) at the C-terminal, responsible for the identification and binding of specific PAMP and DAMP [16]. Nucleotide-binding oligomerization domain (NOD) in the middle is the characteristic of NLR, also known as the NACHT domain, named by the first letter of four known NLR family members [17].The function of NOD prompts NLR molecular aggregation to change its configuration [17]. The N-terminal is the effector domain, mainly composed of caspase recruitment domain (CARD), pyrin domain (PYD), or baculovirus inhibitor of apoptosis protein repeat domain (BIR), which mediates the homologous protein interaction to transmit signals downstream [18]. According to the structural characteristics of the effect domain, several subfamilies can be further divided, including NLRA, NLRB, NLRC, and NLRP [19].

**Figure 1:**
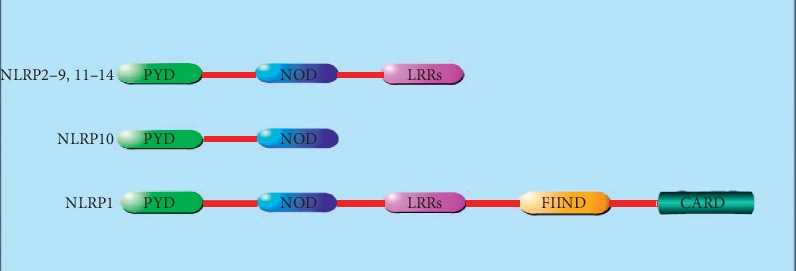
Structure of NLRP in humans. NLRP is the largest subfamily of NLR. All members in NLRP family have the pyrin domain (PYD) at the N-terminal and the nucleotide-binding oligomerization domain (NOD), also named as NACHT domain, in the middle. In addition, most members of NLRP (2–9, 11–14) have leucine-rich repeats (LRRs) at the C-terminal. The NLRP1's C-terminal has the caspase recruitment domain (CARD), function-to-find domain (FIIND), and leucine-rich repeats (LRRs).

**Figure 2:**
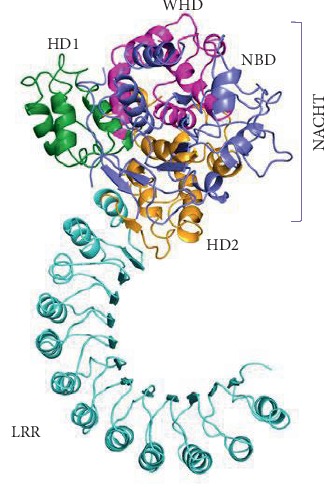
Cryo-EM structure overview. It is a ribbon diagram of the NLRP3 inflammasome with pyrin domains (PYD) deleted. Domains are colour coded in Figure 2. NLRP3 has an N-terminal pyrin domain, which interacts with the adaptor protein ASC via interactions between pyrin domains (PYD); a central adenosine triphosphatase (ATPase) domain known as NACHT, which comprises a nucleotide-binding domain (NBD), helical domain 1 (HD1), winged helix domain (WHD), and helical domain 2 (HD2); and a C-terminal leucine-rich repeat (LRR) domain [21].

NLRP is the largest subfamily of NLR. Currently, 14 kinds of NLRP have been discovered, and NLRP3 has gradually become a “molecule star” (Figure 1) [20]. The structure of NLRP3 was analyzed by Cryo-EM, and the map revealed an earring shape characteristic of NLRs, containing a curved LRR domain and a compact NACHT comprising NBD, HD1, WHD, and HD2 (as shown in Figure 2) [21]. The N-terminus of NLRP3 contains PYD and is mainly expressed in macrophages and peripheral blood leukocytes [22]. NLRP3 can identify and combine with PAMP, such as the MDP, bacterial mRNA, *Listeria monocytogenes*, and *Staphylococcus aureus* [22]. The above events resulted in the conformational change of NLRP3, which exposed it to NOD and promoted oligomerization. Through PYD-PYD interaction, it will recruit apoptosis-related card-like protein (ASC) [23]. Then, NLRP3, ASC, and caspase-1 comprise the NLRP3 inflammasome [24]. ASC recruits procaspase-1 via CARD-CARD interaction, resulting in a conformational change to produce active caspase-1, and cleaving pro-IL-1*β* and pro-IL-18 to the inflammatory cytokines IL-1*β* and IL-18 [24].

### 2.2. Activation of NLRP3

**Figure 3:**
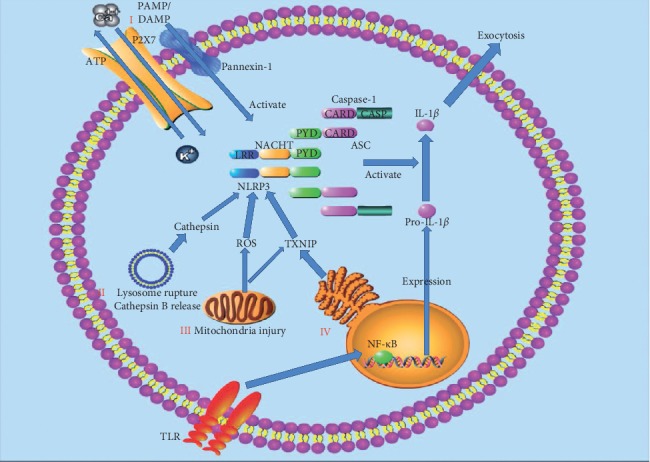
Activation of the NLRP3 inflammasome. Priming is initiated by the TLRs, which recognize and combine the corresponding signals to activate NF-*κ*B at the transcriptional level, facilitating the expression of NLRP3 and various inflammatory precursors, such as IL-1*β* and IL-18 precursors in preparation for the next inflammatory response. NLRP3 is activated by related ligands via ionic flux (I), lysosome rupture and cathepsin B release (II), mitochondrial injury and reactive oxygen species (ROS) generation (III), and endoplasmic reticulum (ER) stress (IV). NLRP3 recruits ASC and procaspase-1 to assemble into the NLRP3 inflammasome. NLRP3 inflammasome which includes mature caspase-1 can promote the activation of proinflammatory mediators such as IL-1*β* and IL-18 and promote the occurrence of inflammatory response.

Production of the NLRP3 inflammasome includes two processes: priming and activation (as shown in Figure 3) [25]. The response is initiated by the TLR, which recognizes and binds the corresponding signals to activate NF-*κ*B at the transcriptional level, facilitating the synthesis of NLRP3 and various inflammatory precursors, such as IL-1*β* and IL-18 precursors, in preparation for the next inflammatory response [26]. NLRP3 is activated by related ligands and then recruits ASC and procaspase-1 to assemble into the NLRP3 inflammasome [27]. NLRP3 inflammasome which includes mature caspase-1 can promote the activation of proinflammatory mediators such as IL-1*β* and IL-18 and promote the occurrence of inflammatory response [27]. The activation mechanism of NLRP3 in the second stage is the focus of the current research, and a variety of mature hypotheses have been formed and supplemented to explain the activation process of NLRP3.

#### 2.2.1. The First Hypothesis: Ion Flow Hypothesis

When cells are damaged or necrotic, ATP generated inside the cell is released to outside of the cell to activate the P2X7 ion channel controlled by ATP on the membrane, causing ion transmembrane migration [28]. Under the continuous stimulation of ATP, P2X7 receptors lead to a large amount of Ca^2+^ and Na^+^ inflow, resulting in the efflux of K^+^ [29]. P2X7R channel opening breaks the intracellular ion balance and makes pannexin-1 as the half channel protein to form pores on the cell membrane. Extracellular ligands (e.g., ATP and LPS) enter the cell, activate NLRP3 inflammatory cells, and promote the secretion and release of IL-1*β* [30]. It has been reported that inflammasomes can be triggered to assemble and recruit procaspase-1, when the intracellular concentration of K^+^ is less than 90 mmol·l^−1^ ([K^+^] < 90 mmol·l^−1^) [31]. Therefore, intracellular low concentration of K^+^ is recognized as a common mechanism to induce activation of the NLRP3 inflammasome [32].

P2X7 receptor activation also promotes calcium influx, and Ca^2+^ mobilization in the NLRP3 inflammasome activation is prevalent but ambiguous [33]. Chu et al. showed that the BAPTA-AM, a Ca^2+^ chelator, inhibits IL-1 formation, suggesting the involvement of Ca^2+^ mobilization in NLRP3 inflammasome activation [34]. The increase in intracellular Ca^2+^ from varieties of Ca^2+^ pool plays a critical role in activation of the NLRP3 inflammasome [35]. The inhibitor of IP3R prevents Ca^2+^ flux and also hinders the NLRP3 activation [36]. Additionally, the entry of Ca^2+^ through Ca^2+^ channels on the plasma membrane is an indispensable approach to the increase in Ca^2+^ in the cytosol, such as P2X7R, TRPM2, and TRPM7 [37]. Beyond the above, the lysosome has ability to release Ca^2+^ in the process of NLRP3 inflammasome activation [38]. The inhibition of the ER or plasma membrane Ca^2+^ channels will abate caspase-1 activation and IL-1*β* secretion in response to NLRP3 stimuli [38]. In spite of abundant studies on Ca^2+^ in NLRP3 activation, the accurate mechanism has not been revealed. It was successfully presented that Ca^2+^ overloading of mitochondria was involved in NLRP3 activation [35]. In conclusion, Ca^2+^ mobilization may play an auxiliary role in NLRP3 inflammasome activation relatively.

#### 2.2.2. The Second Hypothesis: Lysosomal Hypothesis

Some crystals or granular substances such as sodium urate crystals [39], calcium oxalate crystals [40], alum [41], asbestos [42], and *β*-amyloid [43] are phagocytized by phagocytes. The lysosome structure is damaged, and the protease in lysosomes, mainly cathepsin B, is released into the cytoplasm to activate NLRP3 [44]. This hypothesis includes two aspects: lysosome rupture and protease release. First, lysosome rupture undoubtedly plays an important role in the activation of NLRP3, but the exact mechanism is still not fully understood. Hornung et al. found that during the activation of NLRP3, the application of H^+^-ATPase inhibitors inhibits the activation of NLRP3, suggesting that acidic environment was essential in NLRP3 activation [44]. Schorn et al. have proposed that lysosome lysis provides an acidic environment, and a large amount of Na^+^ are released from the lysosome [45]. Increased intracellular osmotic pressure results in excessive intracellular water and decreased intracellular K^+^ concentration, which may be further involved in NLRP3 activation [45]. Second, proteases released from the lysosome are essential in the activation of NLRP3. Studies have shown that inhibitors of cathepsin B significantly inhibit the activation of NLRP3, so cathepsin B is more concerned by the community [45]. It was reported that lysosomal cathepsin B was closely related to the release of IL-1*β*, demonstrating the importance of cathepsin B in the activation of NLRP3 [4♦6]. Similarly, how cathepsin B is involved in the activation of NLRP3 remains unclear.

#### 2.2.3. The Third Hypothesis: ROS and Mitochondria Hypothesis

ROS pathway is often considered as a common pathway for NLRP3 inflammasome assembly since most of the activators induce ROS production and activate downstream of NLRP3 [47]. Mitochondria are the main production sites of ROS. mtROS and mtDNA produced with mitochondrial dysfunction are related to the activation of NLRP3 inflammasome [48]. Nakahira et al. found that mtROS caused by mitochondrial respiratory chain inhibition was crucial in the activation of NLRP3 induced by LPS and ATP [49]. Meanwhile, mtDNA was also released into the cytoplasm, which promoted NLRP3 activation [50]. However, some scholars have questioned whether the inhibition of NLRP3 focuses on the activation of LRP3, or the activation of NLRP3. A study has shown that the priming of NLRP3 is inhibited when antioxidants block mtROS [51]. The direct mechanism of mtROS and NLRP3 still needs to be proved by experiments. In recent years, a study has found that thioredoxin interacting protein (TXNIP) is closely connected with the activation of NLRP3 [52]. TXNIP can be activated by ROS and then induce the generation of ROS in turn [52]. TXNIP is related to the redox of thioredoxin (TRX). When TXNIP binds to TRX, the produced ROS can make TRX oxidized [53]. TXNIP is isolated from the oxidized TRX and binds to the downstream of NLRP3 to participate in NLRP3 activation [54]. TXNIP is also involved in the indirect activation of NLRP3 through NEK7 activation [55]. During mitochondrial injury, cardiolipin in the mitochondrial membrane was also exposed [56]. Iyer et al. found that if the content of cardiolipin decreased, the activation of NLRP3 was also inhibited [57].

#### 2.2.4. The Fourth Hypothesis: Endoplasmic Reticulum Stress Hypothesis

When stressors are applied to cells, they induce misfolded and unfolded proteins to accumulate in the endoplasmic reticulum cavity and disrupt Ca^2+^ balance, which in turn activates unfolded protein response (UPR) and apoptotic signaling pathways, which is called endoplasmic reticulum (ER) stress [58]. ER stress plays a crucial role in enhancing the resistance and adaptability of cells to injury and has an important impact on cell survival [58]. Mekahli et al. found that ER stress activated inflammasomes. Inositol-requiring enzyme 1*α* (IRE1*α*), activating transcription factor-6 (ATF6), and protein kinase R-like endoplasmic reticulum kinase (PERK) are ER stress transmembrane sensors which are involved in ROS production and NLRP3 activation [59]. Studies have proved that IRE1*α* and PERK also indirectly activate NLRP3 by increasing TXNIP [60]. On the other hand, the endoplasmic reticulum is an important intracellular calcium reservoir, and the entry of Ca^2+^ to the mitochondria from the endoplasmic reticulum [61] promotes ROS production, leading to NLRP3 activation.

## 3. NLRP3 Inflammasome and AS

### 3.1. Inflammation in AS

Inflammation runs through the beginning, progression, and complications of atherosclerosis [62]. Atherosclerosis is a complex, multimechanical disease. Inflammatory mediators such as histamine, tumor necrosis factor (TNF), and interleukin-1 (IL-1) can lead to rupture and dysfunction of the vascular endothelium, thus causing a large number of inflammatory cells to gather at the injured situation and migrate into the subcutaneous tissue, inducing the inflammation [63]. The inflammation requires pattern recognition receptors (PRRs) to recognize and combine with pathogen/danger-associated molecular patterns (PAMPs/DAMPs), which can rapidly activate inflammasomes. NLRP3 inflammasome acts on the target tissues and prompts the functional changes of the target tissue to adapt to the harmful environment [64]. Duewell et al. found that cholesterol crystals promoted the secretion of saspase-1 and IL-1 by activating the NLRP3 inflammasome. The literature also reported that mice fed with high-fat diets showed increased caspase-1 expression after 3 weeks, and some papers proved that caspase-1 activation was positively correlated with lipid levels [65]. After oxidative modification, ox-LDLs have a strong effect on AS. Studies have found that ox-LDLs activate the NLRP3 inflammasome through the ROS pathway. Sheedy et al. further found that phagocytosis of ox-LDL promoted the activation of the NLRP3 inflammasome [66]. The above studies show that the NLRP3 inflammasome plays an important role in the process of AS.

### 3.2. NLRP3 Inflammasome in AS Patients

In recent years, several population epidemiology studies have provided indirect evidence for the relationship between the NLRP3 inflammasome/IL-1*β* signaling pathway and AS, as shown in Table 1. The expression of NLRP3 in ascending aorta tissues of patients with coronary artery bypass grafting (CABG) is significantly higher than that of patients without AS, and it is positively correlated with the lesion degree of AS and risk factors of AS [67]. NLRP3 in the aorta was significantly correlated with the severity score of Gensini on the coronary artery [70]. The relevant components in the inflammasome signaling pathway of NLRP3, ASC, caspase-1, IL-1*β*, and IL-18 are highly expressed in human carotid atherosclerotic plaques, while expression in healthy mesenteric arteries is weak [68]. Compared with stable plaques, the levels of NLRP3, ASC, caspase-1, IL-1*β*, and IL-18 in unstable plaques are higher [68]. The expression of NLRP3-mRNA in the plaques of symptomatic AS patients is higher than that of asymptomatic AS patients [69]. Compared with non-CHD (nonchronic heart disease) patients, patients with CHD, especially those with acute coronary syndrome (ACS), have higher levels of mRNA and protein in peripheral blood of the NLRP3 inflammasome [70]. In ACS patients, NLRP3 is positively correlated with the degree of AS by clinical scores and lesion characteristics [70]. This study showed that the baseline concentration of NLRP3 was a promising prognostic index that effectively predicted MACE events through Grace and TIMI risk scores [70].

### 3.3. NLRPP3 Inflammasome in AS-Molded Animals

More and more studies have demonstrated that the NLRPP3 inflammasome is activated in atherosclerosis-molded animals and plays a crucial role in the development of atherosclerosis, as shown in Table 2. Wang et al. found that the expression of NLRP3 inflammasome was increased in ApoE^−/−^ mice fed with a high-fat and high-protein diet [71]. Some studies have shown that plaque stability was increased and the development of atherosclerosis was inhibited after NLRP3 expression was silenced by the NLRP3 shRNA virus [72]. Abderrazak et al. found that NLRP3 gene knockout reduced the area of atherosclerotic plaque in the whole aorta and aortic sinus in ApoE^−/−^ mice fed with a high-fat diet [73]. Arglabin, a plant-derived compound, inhibited the activity of the NLRP3 inflammasome and significantly reduced the production of IL-1*α*, IL-1*β*, and IL-18, reducing the production of proinflammatory mediators to alleviate atherosclerosis [73]. Duewell et al. found that in mice lacking the inflammasome components of NLRP3, the level of IL-18-dependent NLRP3 inflammasome in atherosclerosis caused by cholesterol crystals was reduced, which provides further clear evidence to support the importance of the NLRP3 inflammasome and cholesterol crystals in the development of atherosclerosis [65]. Shen et al. conducted relevant studies that polyunsaturated fatty acids in diet inhibited the activation of the NLRP3 inflammasome, and thus reduced the occurrence of atherosclerosis [76]. It was also reported that NLRP3 inhibitors such as MCC950 applying to ApoE^−/−^ mice after four weeks showed that although the mice body quality, blood sugar, very low-density lipoprotein cholesterol (VLDL-c), low-density lipoprotein cholesterol (LDL-c), high-density lipoprotein cholesterol (HDL-c), triacylglycerol, and total cholesterols had no obvious changes, the area of atherosclerotic plaque decreased significantly, which in turn showed that MCC950 can inhibit the expression of NLRP3 for treatment of atherosclerosis [75]. These results suggest that inhibiting the expression of the NLRP3 inflammasome reduces the development of atherosclerosis. The ox-LDLs [77], cholesterol crystals [65], and other substances induced the activation of the NLRP3 inflammasome and thus promoted the generation of atherosclerosis. Gage et al. found that compared with ApoE^−/−^/caspase-1^−/−^ double knockout mice, the extent of macrophage infiltration and the area of atherosclerotic plaque were significantly reduced in ApoE^−/−^ mice [78]. To further clarify the exact relationship between the NLRP3 inflammasome and atherosclerosis, and the specific mechanism, we need to provide more reliable experimental evidence.

### 3.4. NLRP3 Inflammasome in Cells In Vitro

**Figure 4:**
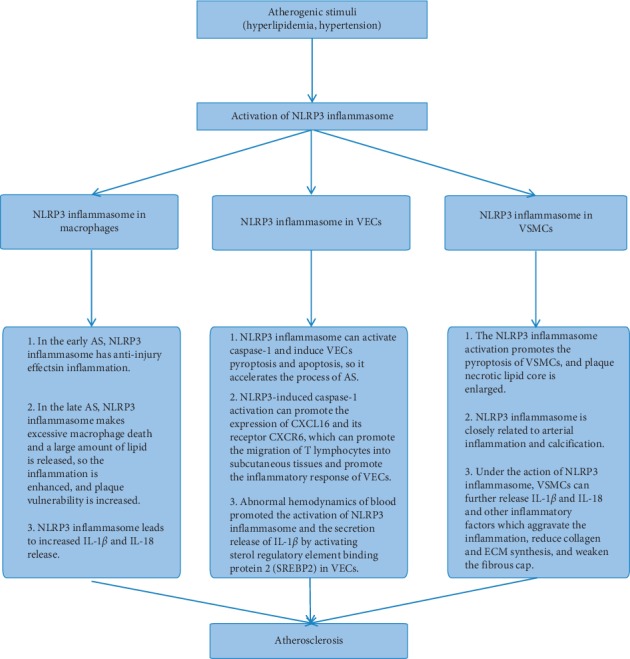
The role of the NLRP3 inflammasome in AS.

In the process of AS, a variety of cardiovascular damage factors can trigger the reaction of inflammatory cells such as macrophages, VECs, and VSMCs through activation of the NLRP3 inflammasome, resulting in a large release of inflammation mediators such as IL-1*β* and IL-18, which further induces the local and systemic inflammatory cascade, promoting the formation, vulnerability, and rupture of plaque. In recent years, the beneficial exploration of regulation of the NLRP3 inflammasome has also provided a new perspective for the treatment of AS (as shown in Figure 4).

#### 3.4.1. NLRP3 Inflammasome and Macrophages

Macrophages play an important role in the early or late plaque formation and plaque rupture in AS [79]. In the early AS, NLRP3 derived from macrophages is involved in the anti-injury reactions of inflammation, which is beneficial to the stability of plaques [80]. Relatively, the NLRP3 inflammasome in the late AS makes excessive macrophage death and a large amount of lipid are released, which contributes to increase in lipid core and plaque vulnerability [80]. In recent years, a number of studies have proved that oxidized low-density lipoprotein (ox-LDL) and cholesterol crystals can activate the NLRP3 inflammasome and caspase-1, inducing macrophages to pyroptosis and leading to increase in the release of IL-1*β* and IL-18 [65]. The above factors prompt the inflammatory response of AS and reduce plaque stability. Regulation of NLRP3 in macrophages is vital in delaying the progress of AS and enhancing the stability of plaque. Silencing the *NLRP3* gene in mice inhibited the occurrence of inflammatory response, which slowed down the process of AS. Reducing the lipid core within the plaque improves plaque stability [72]. Also, specifically silencing mouse bone marrow *caspase-1/11* gene can significantly reduce the necrotic lipid core of plaque and enhance plaque stability [81]. Studies have found that MCC950, an NLRP3 inflammation inhibitor, can significantly improve the stability of mouse platelets because it inhibits the inflammatory response of macrophages [82]. MCC950 can also inhibit the transformation of macrophages into foam cells by inhibiting ox-LDL uptake and increasing cholesterol outflow, and thus the progression of AS is controlled [82].

#### 3.4.2. NLRP3 Inflammasome and VECs

VECs comprise simple squamous epithelium, which is located in the inner layer of the vascular chamber and has a direct contact with the blood [83]. The dysfunction of VECs is an important link in the formation and development of AS [84]. Recent studies have shown that a variety of damage factors in AS can cause inflammatory response of vascular endothelial cells, and ox-LDL can promote NLRP3 inflammatory response through ROS mechanism, activate caspase-1, and induce heat shock of vascular endothelial cells [85]. Nicotine, as the most common risk, activates the NLRP3 inflammasome to promote the inflammatory response and even cell apoptosis of VECs, so it accelerates the process of AS [86]. The cytokines such as IL-1*β*, IL-18, *P*-selectin, intercellular adhesion molecule-l (ICAM-l), and vascular cell adhesion molecule-1 (VCAM-1) are increased in the inflammation of AS, which trigger the adhesion of the mononuclear phagocyte system and make AS deteriorate [87]. In addition, NLRP3-induced caspase-1 activation increases the expression of CXCL16 and its receptor CXCR6, which promotes the migration of T lymphocytes into subcutaneous tissues and promotes the inflammatory response of VECs [88]. It was found that hemodynamic abnormalities promoted the activation of NLRP3 and the secretion and release of IL-1*β* in human umbilical vein endothelial cells by activating sterol regulatory element binding protein 2 (SREBP2) [89].On the contrary, Yang et al. found that proanthocyanidin B2 inhibited the activation of the NLRP3 inflammasome in LPS-induced HUVECs by downregulating reactive oxygen species (ROS) level, and the activity of caspase-1 and IL-1*β* level was reduced [90]. Mangiferin reduces ROS level in the endothelial cells to inhibit IRE1*α* phosphorylation and reduce ER stress [90, 91]. Thioredoxin interacting protein (TXNIP) expression was impeded, which inhibited NLRP3 activation and IL-1*β* release [91]. Regarding the NLRP3 signaling pathway as the target, melatonin can inhibit the pyroptosis of VECs through the MEG3/miR-223/NLRP3 signaling axis, and its substitute is expected to provide a new prospect for the control of AS [92]. Microrna-30c-5p inhibits NLRP3-induced inflammation in VECs via F0X03, which provides a further step forward for the prevention and treatment of AS [93].

#### 3.4.3. NLRP3 Inflammasome and VSMCs

VSMCs are important cells of the middle membrane in coronary arteries [94]. In the early stage of AS, the activated VSMCs have a strong ability of proliferation and migration, which migrate from the middle membrane to the inner membrane [95]. By secreting an extracellular matrix, the fibrous cap is stabilized, which plays an important role in preventing plaque rupture. However, in the late stage of AS due to a large amount of lipid accumulation in the plaque, cholesterol activates a variety of proinflammatory genes in vascular smooth muscle cells, leading to the activation of NLRP3 inflammatory response in vascular smooth muscle cells, exacerbating the inflammatory response, and eventually resulting in vascular smooth muscle cells and plaque necrotic lipid nuclear heat sags [95]. Studies have found that calcium crystals make the NLRP3 inflammasome activated. It was reported that intracellular mRNA levels of NLRP3, ASC, and caspase-1 were increased when *β*-glycerophosphate (*β*-GP) induced the primary rat aorta VSMCs to get crystallization on calcium. The level of IL-1*β* and VSMCs calcification was inhibited after the NLRP3 was silenced. At the same time, in the calcified tissue of the human artery, the levels of mRNA were significantly upregulated, and caspase-1 activity was increased [96]. It is suggested that the NLRP3 inflammasome is closely related to arterial inflammation and calcification. In a study by Usui et al., the *a*-smooth muscle actin (*α*-SMA), as a marker of VSMCs, was detected by the immunohistochemical method, and compared with ApoE^−/−^ mice, the number of VSMCs at the inner membrane decreased significantly in ApoE^−/−^/caspase-1^−/−^ mice plaques [97]. Absent in melanoma- (AIM2-) related pattern recognition receptor can activate caspase-1 through the NLRP3 pathway, and then it mediates the inflammatory response of VSMCs by cutting GSDMD [98]. Under the action of the NLRP3 inflammasome, VSMCs also further released IL-1*β* and IL-18 and other inflammatory factors, which aggravated the inflammation, reduced collagen and ECM synthesis, and weakened the fibrous cap. Therefore, increased vulnerability of plaque lead to plaque erosion and rupture.

## 4. Therapy Targeting NLRP3 for AS

Because inflammatory response and NLRP3 inflammasome play an important role in the development of atherosclerosis, the NLRP3 inflammasome as the therapeutic target has become a hot topic in the research of atherosclerotic drugs. Emerging evidence suggests that the NLRP3 inflammasome could be considered as the potential therapeutic target for atherosclerosis, as shown in Table 3.

### 4.1. Natural Medicine and the Treatment for AS

Artemisinin is a natural peroxide lactone compound extracted from the plant *Artemisia annua* which showed vascular protection function. Artemisinin (50 or 100 mg/kg) can effectively improve formation and proliferation of foam cells and promote fibrosis in the intima of the aorta. It was reported that artemisinin inhibited inflammatory responses through the AMPK/NF-*κ*B-NLRP3 pathway in macrophages [99].

Pretreating with rosmarinic acid (RA), the volume of nicotine-induced C-reactive protein (CRP) [86] will be dropped in VSMCs. In addition. RA also inhibited the activation of pyrin domains in the NLRP3 inflammasome and reduced the production of ROS after nicotine was involved in VSMCs. *In vivo* experiments suggested that RA played a protective role in nicotine-induced atherosclerosis *via* inhibiting the axis of ROS-NLRP3-CRP, and therefore RA was a potentially effective treatment for atherosclerosis, especially in smokers [100].

Curcumin significantly decreased the expression of NLRP3, caspase-1, and IL-1*β* in phorbol 12-myristate 13-acetate-(PMA-) induced macrophages. Curcumin is also partially involved in the phosphorylation of TLR4, MyD88, and I*κ*B-*α*, as well as activating NF-*κ*B. Therefore, curcumin inhibited NLRP3 inflammasome expression in PMA-induced macrophage by inhibiting TLR4/MyD88, NF-*κ*B, and P2X7R [101].

Atherosclerosis is a chronic inflammatory disease mainly caused by the accumulation of cholesterol and the formation of cholesterol crystals (CCs) in the subcutaneous tissue. These CCs promote the development of the disease by activating the NLRP3 inflammasome and triggering a complex inflammatory response. Recently, many studies focused on whether ursodeoxycholic acid (UDCA) affected the formation of vascular CCs. It was reported that UDCA induced intracellular CC dissolution in macrophages, reducing the secretion of IL-1*β*. In summary, most of the data suggested that UDCA reduced CCs and attenuated NLRP3-dependent inflammation by increasing cholesterol solubility in mice [102].

It was observed that berberine suppressed IL-1*β* secretion in macrophages. In addition, Jiang et al. demonstrated that berberine reduced the activation of the NLRP3 inflammasome via the ROS-dependent pathway, which provided the evidence for the hypothesis that berberine alleviated NLRP3 inflammasome activation and reduced IL-*β* secretion from macrophages, showing an important therapeutic target in atherosclerosis therapy [103].

Dihydromyricetin (DHM) is a kind of natural flavonoids with antioxidant, anti-inflammatory, and other biological activities. In a study, palmitic acid (PA) treatment resulted in caspase-1 activation, lactate dehydrogenase (LDH) release, and positive-staining of propidium iodide in HUVECs. PA can promote the maturation and release of proinflammatory cytokines especially IL-1*β* by elevation of intracellular ROS and mtROS. In addition, transfection with NLRP3 inhibitors or treatment with NLRP3 siRNA effectively inhibited PA-induced pyroptosis, while pretreatment with total ROS or mtROS scavenger attenuated NLRP3 inflammasome activation and subsequent pyroptosis. DHM inhibited PA-induced high-temperature cell death by increasing cell viability and reducing caspase-1 and IL-1*β* release to improve cell membrane integrity. This study showed that DHM pretreatment significantly reduced intracellular ROS and mtROS levels and activated the Nrf2 signaling pathway [104]. In summary, these results suggested that the Nrf2 signaling pathway was obviously partially involved in the DHM-mediated improvement of PA-induced vascular events, suggesting the potential medicinal value of DHM against immune/inflammation-related diseases such as atherosclerosis.

Trimethylamine N-oxide (TMAO) is associated with endothelial dysfunction in atherosclerosis, a cardiovascular disease induced by vascular inflammation. TMAO induces scavenger receptors, adhesion molecules, and other genes associated with atherosclerosis in VECs. Apigenin is rich in celery and parsley, which prevents endothelial cells from artery injury [105]. Apigenin can reverse the transcription of LOX-1, SREC, SR-PSOX, NLRP3, TXNIP, VCAM-1, ICAM-1, and MCP-1, as well as the translation of LOX-1, adhesion molecule ICAM-1, and NLRP3 inflammasome. Apigenin also inhibited leukocyte adhesion and acetylated LDL uptake [105].

### 4.2. Clinical Medicine and the Treatment for AS

To evaluate the activation of inflammasomes in monocytes of patients with acute coronary syndrome (ACS) and the short-term oral administration of colchicine [113] (a recognized anti-inflammatory drug shown in clinical studies to have a protective effect on the heart), ACS patients (*n* = 21) were randomly divided into the oral colchicine group and the untreated group and compared with the untreated healthy control group (*n* = 9). Treatment for ACS with colchicine significantly reduced the levels of caspase-1 and IL-1*β* in the cells [106, 107].

Statins are very important in prevention and treatment for cardiovascular disease by inhibiting cholesterol synthesis. However, the beneficial effect of statins in cardiovascular disease may also be due to their role as anti-inflammatory mediators. Atorvastatin, a 3-hydroxy-3-methyglutaryl coenzyme A (HMG-CoA) inhibitor, significantly reduced the expression of NLRP3, caspase-1, and IL-1*β* in PMA-induced THP-1 cells. In addition, the NF-*κ*B inhibitor decreased the expression of inflammatory mediators in inflammatory cells. It was suggested that the activation of the NF-*κ*B pathway was involved in the regulation of the NLRP3 inflammasome [108, 109]. Therefore, atorvastatin plays an anti-inflammatory role by inhibiting the PMA-induced THP-1 monocyte via the TLR4/MyD88/NF-KB pathway. *In vitro* and *in vivo* treatment with simvastatin resulted in significantly lower expression levels in response to stimulation with CCs. Simvastatin inhibited expression of IL-1*β*, peripheral blood mononuclear cells (PBMCs), and CCs and then had protective effect on patients with cardiovascular disease [114].

Glucose cotransporter 2 (SGLT2) inhibitor has a good effect on glucose and fat metabolism, and it partially reverses the formation of atherosclerosis through inhibiting the infiltration of macrophages and enhancing the stability of the plaques. Dapagliflozin may have therapeutic abilities for diabetic atherosclerosis induced by a high-fat diet, and these benefits may depend on macrophages inhibiting IL-1*β* secretion through the ROS-NLRP3-caspase-1 pathway [110].

Metformin promotes the secretion of adenosine monophosphate-activated protein kinase (AMPK) and protein phosphatase 2A (PP2A). Data showed that metformin reduced the expression of NLRP3 and inhibited the activation of the NLRP3 inflammasome in ox-LDL-stimulated macrophages through increasing the expression of AMPK and PP2A [111]. Other *in vitro* experiments suggested that high glucose induced the accumulation of ROS and activated the NLRP3 inflammasome, which was significantly inhibited after treatment with metformin or the antioxidant N-acetyl-l-cysteine. In addition, the inhibitor complex C of AMPK hindered the anti-inflammatory effect of metformin, suggesting that metformin inhibited the high-glucose-induced NLRP3 inflammasome through AMPK activation. Glucose decreased the expression of TRX and increased the expression of TXNIP, in which metformin was also reversed. Metformin also inhibited the activation of the NLRP3 inflammasome in ApoE^−/−^ mice and inhibited atherosclerosis in diabetes [115].

### 4.3. Other Treatments for AS

Via increasing cholesterol excretion, the dietary fiber (DF) to reduce the risk of atherosclerosis may occur through many mechanisms. Although macrophages are essential for lipid clearance, excessive uptake of cholesterol crystals (CCs) by these cells still induces the activation of the NLRP3 inflammasome and formation of foam cells. Therefore, the study investigated whether the water-soluble DF of chayote regulated the CCs in macrophage-like THP-1 cells. The health benefits of dietary fiber may exceed its physical properties for the gastrointestinal tract. Studies have evaluated the antiatherogenic effects of oat fiber and wheat bran fiber and explored their potential anti-inflammatory mechanisms. Animal experiments, pathology, and biological analysis have shown that cereal fiber can reduce the inflammatory response and atherosclerosis in ApoE^−/−^mice. These effects are evident in oat fiber, which may be mediated by specific inhibition of the NLRP3 inflammasome pathway [112].

Dietary polyunsaturated fatty acids (PUFAs) reduce macrophage inflammation and delay the progress of atherosclerosis, but the accurate mechanisms are poorly understood. Through animal experiments, it was concluded that dietary PUFAs could reduce atherosclerosis by activating macrophage autophagy and inhibiting the activation of the NLRP3 inflammasome [112].

Melatonin has been reported to have a number of anti-inflammatory properties, shown to be effective against AS. Melatonin decreased expression of genes associated with the aortic endothelium, including NLRP3, ASC, cleaved caspase-1, NF-*κ*B/GSDMD, GSDMD-N, and IL-1*β*. Through the MEG3/miR-223/NLRP3 axis [92], it can prevent apoptosis of endothelial cells in atherosclerosis. Current studies have shown that melatonin prevented the progression of atherosclerosis by inducing mitophagy and attenuating the activation of the NLRP3 inflammasome via the Sirt3/FOXO3a/Parkin signaling pathway [74].

## 5. Conclusion

“Inflammation theory” plays an important role in the development of atherosclerosis. NLRP3 inflammasome is pivotal in the stability of plaques owing to the ion flow, lysosome rupture, and ROS and endoplasmic reticulum stress mechanism, and then the NLRP3 inflammasome activation will produce mature inflammatory mediators such as IL-1*β* and caspase-1 which participate in the development of atherosclerosis. Most studies have proved that targeting at the initiation and activation of the NLRP3 inflammasome can effectively delay the process of atherosclerosis, and thus reduce the hospitalization rate of patients. However, clinical data are insufficient and some mechanisms about how the NLRP3 inflammasome participates in AS has not been explained clearly. Using drugs targeting at the NLRP3 inflammasome to treat atherosclerosis is promising, but it also needs further pharmacological studies to verify the efficacy and further experimental epidemiological studies to ensure the safety. In addition, to make the drugs widely used clinically, evidence-based medicine play an indispensable role.

## Figures and Tables

**Table 1:** The role of NLRP3 inflammasome in AS patients.

Study type	Subjects	Effect of NLRP3 inflammasome	References
Clinical	The ascending aorta tissues of patients with CABG	The expression of NLRP3 is higher than that of patients without AS, which is positively correlated with the lesion degree of AS.	[67]
	Carotid atherosclerotic plaques in human	NLRP3, ASC, caspase-1, IL-1*β*, and IL-18 are highly expressed, especially in unstable plaques.	[68]
	The plaques of symptomatic AS patients	The expression of NLRP3-mRNA is higher than that of asymptomatic AS patients.	[69]
	The peripheral blood of CHD patients with ACS	The patients with ACS have higher levels of NLRP3 inflammasome.	[70]

CABG: coronary artery bypass grafting; CHD: chronic heart disease; ACS: acute coronary syndrome.

**Table 2:** The effect of NLRP3 inflammasome inhibitors in AS-molded animals.

Study type	Subjects	Inhibitors	Effect	References
In vivo	ApoE^−/−^ mice fed on a high-fat and high-protein diet	NLRP3 shRNA virus suspension (1.75 × 108 Tfu, 20 *μ*L)	NLRP3 inflammasome inhibited; plaque stability increased; development of AS inhibited	[72]
	ApoE^−/−^ mice fed on a high-fat diet	Arglabin	The activity of NLRP3 inflammasome inhibited; the production of proinflammatory mediators reduced	[73]
	ApoE^−/−^ mice	Polyunsaturated fatty acid (vegetable oil and animal oil added into diets for additional 8–16 weeks)	The activation of NLRP3 inflammasome inhibited; the occurrence of AS reduced	[74]
	ApoE^−/−^ mice	MCC950 (10 mg/kg)	The area of atherosclerotic plaque decreased significantly	[75]

**Table 3:** The therapy targeting NLRP3 inflammasome for AS.

Therapy type	Medicine	Chemical structure	Effects and mechanisms	References
Natural medicine	Artemisinin (50, 100 mg/kg)	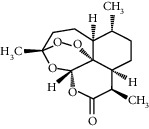	Vascular protection: the formation and proliferation of foam cells improved; the fibrosis in the intima of aorta promoted Inflammation inhibition: targeting the AMPK/NF-*κ*B-NLRP3 pathway	[99]
	Rosmarinic acid (100 *μ*M)	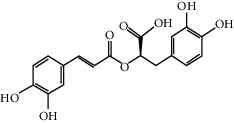	Playing a protective role in nicotine-induced AS via inhibiting the axis of ROS-NLRP3-CRP	[100]
	Curcumin (0–100 *μ*M)	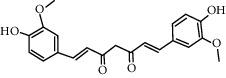	Inhibiting NLRP3 inflammasome in PMA-induced macrophage by inhibiting TLR4/MyD88, NF-*κ*B, and P2X2R	[101]
	UDCA (20 *μ*g/ml)	—	Attenuating NLRP3-dependent inflammation: reducing CCs; increasing cholesterol solubility	[102]
	Berberine (75 *μ*M)	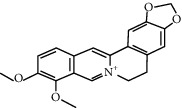	Alleviating NLRP3 inflammasome activation and reducing IL-1*β* secretion	[103]
	DHM (0.1, 0.5, and 1 *μ*M)	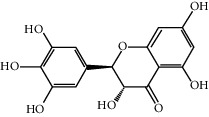	Antioxidant and anti-inflammatory activities: ROS reduced; the release of caspase-1 and IL-1*β* reduced	[104]
	Apigenin (50 *μ*M)	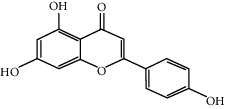	Endothelium protection: reversing the expression of adhesion molecule ICAM-1 and NLRP3 inflammasome	[105]
Clinical medicine	Colchicine (1 mg followed by 0.5 mg 1 hour later)	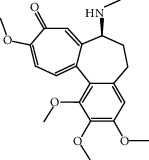	The levels of caspase-1 and IL-1*β* reduced	[106, 107]
	Statins (atovastatin 0–40 *μ*M)	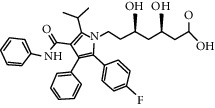	Inhibiting cholesterol synthesis; anti-inflammatory function	[108, 109]
	Dapagliflozin (1.0 mg/kg/d)	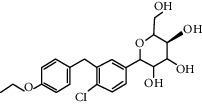	Inhibiting IL-1*β* secretion through the ROS-NLRP3-caspase-1 pathway	[110]
	Metformin (300 mg/kg/d, drinking water)	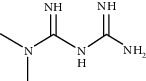	Anti-inflammatory function: reducing NLRP3 expression; inhibiting NLRP3 activation	[111]

Others	Dietary fiber	—	Antiatherogenic effects; anti-inflammatory effects	[112]
	Dietary PUFAs	—	Activating macrophage autophagy; inhibiting the activation of NLRP3 inflammasome	[112]
	Melatonin (20–2000 *μ*M)	—	Anti-inflammatory effects; preventing apoptosis of endothelial cells; attenuating NLRP3 inflammasome activation	[74]

AMPK: adenosine monophosphate-activated protein kinase; CRP: C-reactive protein; PMA: phorbol 12-myristate 13-acetate; UDCA: ursodeoxycholic acid; CCs: cholesterol crystals; DHM: dihydromyricetin; TMAO: trimethylamine N-oxide; PUFAs: dietary polyunsaturated fatty acids.
